# E-cigarette use among adults in southern Iran: a qualitative study

**DOI:** 10.1186/s12889-026-27917-w

**Published:** 2026-06-09

**Authors:** Yaser Rastegar, Hadi Eshaghi Sani Kakhaki, Parniya Abolghaseminejad, Sara Dadipoor, Asiyeh Yari, Ebbie Kalan

**Affiliations:** 1 Department of Social Sciences, University of Hormozgan, Bandar Abbas, Iran; 2 Department of Occupational Medicine, School of Medicine, Hormozgan University of Medical Sciences, Bandar Abbas, Iran; 3 Student in Health Education and Promotion, Department of Health Education and Promotion, School of Public Health, Tehran University of Medical Sciences, Tehran, Iran; 4 Tobacco and Health Research Center, Hormozgan University of Medical Sciences, Bandar Abbas, Iran; 5 Noncommunicable Diseases Research Center, Fasa University of Medical Sciences, Fasa, Iran; 6 Department of Behavioral and Community Health, School of Public Health, University of Maryland, College Park, MD USA

**Keywords:** Electronic cigarette, Vaping, Socio-cultural factors, Nicotine dependence, Thematic analysis, Iran

## Abstract

**Background:**

espite Iran’s ban on e-cigarettes since 2012, their use continues among adults due to complex individual, social, and cultural factors. Qualitative evidence exploring these determinants in Iran remains limited. This study examined the motivations and experiences of adult e-cigarette users aged 19–52 years in southern Iran.

**Methods:**

A qualitative study using thematic analysis, guided by Braun and Clarke’s six-step framework, was conducted in 2024. Participants were recruited using a combination of purposive and snowball sampling techniques and Data were gathered through semi-structured interviews with 45 current (past 30 days) e-cigarette users residing in Bandar Abbas. Two researchers independently coded and analyzed the data, employing strategies to enhance trustworthiness, including investigator triangulation and member checking.

**Results:**

In this study, 45 people (37 men and 8 women) with a mean age of 25.8 years and an age range of 19 to 52 years participated. Our analysis identified six key themes: (1) Sensory and experiential appeal (e.g., diverse flavors, pleasant sensations, modern design), (2) Emotional regulation and stress alleviation, (3) Social contexts and normalization (e.g., peer pressure, familial acceptance, influencer marketing), (4) Perceived practical benefits and misconceptions (e.g., perceived harm reduction, use as a smoking cessation aid), (5) The role of the tobacco industry (e.g., product design, marketing strategies, and availability), and (6) Physical and psychological dependence. In our analysis, social contexts and normalization were the most important contexts for e-cigarette use, given the target group population.

**Conclusions:**

E-cigarette use among adults aged 19–52 years in Bandar Abbas is driven by a complex interplay of sensory appeal, emotional regulation needs, social normalization, widespread safety misconceptions, and aggressive tobacco industry marketing. Despite Iran’s ban, use persists and is reinforced by physical and psychological dependence, peer influence, and limited healthy recreational alternatives. Culturally tailored, multi-level interventions are urgently needed, including evidence-based public education to counter misconceptions, strict regulation of industry advertising and accessibility, and the development of alternative stress-coping resources, especially for younger users.

**Supplementary Information:**

The online version contains supplementary material available at 10.1186/s12889-026-27917-w.

## Introduction

Electronic cigarettes (e-cigarettes or vapes)are battery-operated devices available in various shapes and sizes. Typically containing nicotine, they have gained substantial global popularity over the past decade [1, 2]. Globally, over 10% of high school and university students are current e-cigarette users(used at least once in the past 30 days), with 22% reporting having tried them at least once [1]. Data from the US Centers for Disease Control and Prevention (CDC) indicate a rise in e-cigarette use among American adults, from 4.5% in 2019 to 6.5% in 2023, with the highest prevalence observed among young adults aged 18–24 [3]. The World Health Organization (WHO) has warned that the aggressive marketing of these products threatens to undermine tobacco control efforts, particularly among adolescents; reports indicate that up to 6% of adolescents aged 13–15 in some countries are e-cigarette users [4]. This rising trend, despite a decline in some Western nations, presents significant challenges for public health policy-making [5].

Key motivations for vaping include the availability of appealing flavors, the intention to quit conventional smoking, and various social-identity factors (e.g., peer influence, social acceptance, and self-image) [6, 7]. A qualitative study in Australia revealed that, beyond the perceived physical relief, users often engage in identity formation within a distinct vaping subculture [7]. Conversely, adolescents in the US often find generic anti-vaping campaigns ineffective, calling for more authentic and relatable messaging that reflects their lived experiences [8]. While some research points to the potential role of e-cigarettes in smoking cessation, a growing body of evidence highlights associated physical health risks, nicotine dependence, and prevalent misconceptions regarding their safety [9]. Medical professionals in Turkey, for instance, emphasize the respiratory and cardiovascular risks, noting that e-cigarettes can reinforce nicotine addiction [9]. Features such as discreet use (due to the lack of persistent odor or ash) and the ability to vape frequently further exacerbate the potential for dependence [9].

The nicotine in e-cigarettes is a toxic substance, posing risks through ingestion, inhalation, or dermal/ocular exposure. Alarmingly, over 80% of reported poisoning cases involve children under five years of age [10]. Cases of seizures have also been documented among adolescent and young adult users [11]. Nicotine dependence is characterized by craving, failed attempts to quit, and impaired academic and social functioning [12]. Withdrawal symptoms commonly include irritability, anxiety, depressive mood, and sleep disturbances [12]. Furthermore, vaping is increasingly associated with poor mental health outcomes, including anxiety and depression, creating a cyclical relationship that sustains dependence [12–19]. It is well-established that quitting tobacco leads to reductions in anxiety and depression and an improved quality of life [20, 21]. Critically, no form of tobacco product, including e-cigarettes, is safe. Their use is strongly discouraged for adolescents, pregnant women, and never-users of tobacco [10]. Although switching from combustible cigarettes to e-cigarettes may reduce harm for some adult smokers, it is crucial to note that no e-cigarette product has been approved by the U.S. Food and Drug Administration (FDA) as a smoking cessation aid [3, 10].

Despite Iran’s comprehensive 2012 ban on the sale, production, importation, and distribution of e-cigarettes, their use among young people, particularly in southern regions, continues to rise alarmingly [22]. Anecdotal evidence [23] reveals a thriving underground market where e-cigarettes are readily available through online platforms and informal retail channels, accessible to adults and children alike, especially young women. This paradox underscores a critical public health challenge: while Iran’s tobacco-related research has traditionally focused on conventional cigarettes and waterpipe (aka hookah) consumption, e-cigarettes have emerged as a new and largely unexamined threat. Despite their rapid proliferation among Iranian youth, no comprehensive study has investigated the individual, social, cultural, and psychological determinants driving e-cigarette uptake in this unique context. This significant research gap has left public health policymakers and planners without the evidence needed to develop culturally relevant preventive interventions, control use behaviors, and mitigate associated physical and psychological harms.

In Iran, research on e-cigarette use has largely overlooked key social–cultural, psychological, and identity-related determinants, such as peer norms, gendered meanings, stress coping, and identity construction. Existing evidence is mainly quantitative and context-insensitive. By examining e-cigarette use within a setting of legal prohibition, informal markets, and strong socio-cultural norms, this qualitative study offers novel insights. Therefore, this qualitative study employs a thematic analysis approach to explore the perspectives, motivations, and lived experiences of factors influencing e-cigarette users among adults aged 19–52 years in the socio-cultural context of southern Iran, specifically in Bandar Abbas. By understanding how and why e-cigarettes have penetrated this banned market and what drives youth engagement despite legal prohibition(Considering that the use of e-cigarettes by people under the age of 18 is prohibited in Iran), the findings are expected to inform the development of targeted preventive interventions, evidence-based public health policies, and effective educational programs tailored to the needs of this vulnerable population.

## Methods

### Study design and population

This study, conducted in 2024, employed a qualitative research design, specifically thematic analysis, to identify the factors influencing the tendency to use e-cigarettes. Thematic analysis is recognized as a flexible and robust method for identifying, analyzing, and reporting patterns (themes) within qualitative data [24]. This approach facilitates the discovery and description of recurring and significant themes, making it well-suited to address the research questions of this study, which aimed to understand the experiences and perspectives of the participants.

### Participants

Data were collected through semi-structured interviews with 45 residents of Bandar Abbas. The age range of participants was 19 to 52 years, and both male and female groups were included in the study, with the majority of participants being male. Inclusion criteria were: [1] being 18 years or older [2], current or former use of e-cigarettes within the past year [3], residence in Bandar Abbas, and [4] willingness to participate and share personal experiences. Individuals who had never used e-cigarettes or were unwilling to provide informed consent were excluded from the study. Participants were recruited using a combination of purposive and snowball sampling techniques to ensure the inclusion of individuals with relevant and rich experiences related to e-cigarette use. Purposive sampling was initially employed to select participants with diverse characteristics relevant to the study objectives, including gender, age, frequency of e-cigarette use (occasional vs. regular users), and type of device used. The initial participants were identified through local community contacts and health-related social networks and were selected based on their ability to provide rich and varied experiences related to e-cigarette use. Subsequently, snowball sampling was used, whereby initial participants were asked to refer other individuals who met the inclusion criteria and differed from them in key characteristics. This approach facilitated access to a broader and more diverse group of participants. To ensure maximum variation and enhance the credibility of the findings, efforts were made to include participants with different sociodemographic backgrounds and usage patterns. Recruitment continued until data saturation was achieved, meaning that no new themes or concepts emerged from additional interviews. The study received approval from the Ethics Committee of Hormozgan University of Medical Sciences (Ethics Code: IR.HUMS.REC.1402.324).

### Data collection

Prior to the interviews, the study objectives were explained to all participants, and written informed consent was obtained. Participants were assured of the confidentiality of their information, that their identities would not be disclosed in any reports, and that they could withdraw from the study at any stage without any negative consequences. The interviews were conducted in person, with each session lasting 30–70 min. All interviews were audio-recorded with the participants’ permission and subsequently transcribed verbatim. The English version of the interview was uploaded as a supplementary file.

The researchers carefully followed ethical guidelines, which included building trust, obtaining informed consent for recording, maintaining participant anonymity through the use of pseudonyms, ensuring data confidentiality, and guaranteeing the right to withdraw. It is noteworthy that all these procedures were implemented based on a formal agreement between the researchers and the participants.

### Thematic analysis process

The thematic analysis was conducted based on Braun and Clarke’s six-phase approach [24], which provided the conceptual framework for interpreting the data. The six phases included:

1. Familiarization with the Data: Researchers immersed themselves in the transcripts to gain a deep understanding of the content, taking preliminary notes and highlighting key segments.
2. Generating Initial Codes: Meaningful portions of the data were identified and conceptually labeled with descriptive codes to capture core ideas.
3. Searching for Themes: Related codes were grouped to form potential themes that reflected overarching patterns in the data.
4. Reviewing Themes: Potential themes were examined for coherence and distinctness, leading to the refinement, merging, or splitting of themes.
5. Defining and Naming Themes: Each theme was clearly defined and given a descriptive name to accurately represent its content, ensuring relevance to the research questions.
6. Producing the Report: Findings were structured according to the final themes, with thematic descriptions and illustrative quotes providing depth and clarity.

This section presents the conceptual framework for the thematic analysis, while the practical coding procedure including line-by-line coding, merging codes, use of MAXQDA 2022, independent coding by two researchers, initial inter-rater agreement assessment through side-by-side code comparison.

### Trustworthiness

To ensure the trustworthiness of this study, we applied criteria proposed by Lincoln et al. [25], focusing specifically on credibility and confirmability. Credibility was enhanced through investigator triangulation, whereby both data collection and coding/analysis were conducted independently by two researchers whose analyses were systematically compared to achieve consistency and coherence, thereby minimizing discrepancies and contradictions in the identified themes. Confirmability was established through member checking, in which emergent concepts and themes were presented to participants throughout the analysis process. Through subsequent dialogue, we verified interpretations and achieved maximum confirmability, ensuring that findings accurately reflected participants’ perspectives rather than researcher bias.

### Data analysis

All interview data, after verbatim transcription, were imported into MAXQDA 2022. The coding was conducted line by line, following the conceptual framework of Braun and Clarke:

- Open coding: Initial codes were generated openly from the data.
- Initial inter-rater agreement: Before final consensus, both researchers independently coded a subset of transcripts and compared their codes side by side to quantify agreement and identify discrepancies.
- Code comparison and merging: Codes were gradually compared and combined, forming main categories and sub-categories. Discrepancies between coders were resolved through discussion and consensus.
- Data saturation: Analysis continued until no new codes or concepts emerged from the final interviews.

### Ethical considerations

All interviews were conducted in person and in public places. The potential risks of the interviews were minimal and mainly related to discussing personal experiences with e-cigarette use; to minimize any discomfort, the study objectives were explained in advance, participants were informed of the right to withdraw at any stage, and were assured that their information would remain confidential. Participants did not receive any financial compensation, and their participation was voluntary. All interviews were conducted in Persian and then translated into English for reporting. All these measures were carried out in accordance with the ethical guidelines and with the approval of the Ethics Committee of Hormozgan University of Medical Sciences (Ethics Code: IR.HUMS.REC.1402.324).

## Results

A total of 45 participants were included in the study, comprising 37 males and 8 females. The mean age of the interviewees was approximately 25.8 years, with an age range of 19 to 52 years. Regarding educational attainment, 26 participants held a bachelor’s degree, 3 held a master’s degree, and 16 had a high school diploma. In terms of employment status, 27 were employed and 18 were unemployed. These data indicate diversity in educational and occupational status, with a majority of male participants and a significant proportion holding bachelor’s degrees and being employed. Among all, 0 participants used only cigarettes, 33 participants used only e-cigarettes, and 12 participants used both.

Our qualitative analysis revealed six main categories: (1) Sensory and Experiential Appeal (2), Emotional Regulation and Stress Relief (3), Social Contexts and Normalization (4), Perceived Practical Benefits and Misconceptions (5), The Role of the Tobacco Industry, and (6) Physical and Psychological Dependence (Table 1). Overall, the findings highlight several novel and context-specific dimensions of e-cigarette use. Beyond commonly reported factors such as flavor appeal and stress reduction, this study reveals the influential role of family acceptance within a cultural context where hookah use is socially normalized. Additionally, limited recreational opportunities emerged as an important structural factor shaping vaping behaviors. The findings further demonstrate how social media influencers, device aesthetics, and informal market dynamics contribute to the normalization and desirability of e-cigarette use, reinforcing vaping as both a social and cultural practice rather than solely an individual choice.

**Table 1:** Categories and Sub-categories

	Category	Sub-categories
1	Sensory and Experiential Appeal	Curiosity and Novelty
		Pleasant Physical Sensations
		Pleasant Flavors and Flavor Variety
		Playing with Vapor/Smoke
2	Emotional Regulation and Stress Relief	Coping with Negative Emotions
		Accompanying Intense Emotions
3	Social Contexts and Normalization	Peer Pressure and Modeling
		Social Acceptance and Normalization
		Vaping as a Status Symbol
		Family Acceptance and Modeling
		Lack of Recreational Spaces
4	Perceived Practical Benefits and Misconceptions	Less Nicotine
		Vapor Instead of Smoke
		Tool for Smoking Cessation
5	The Role of the Tobacco Industry	Portability
		Affordability
		Aesthetic Appeal
6	Physical and Psychological Dependence	Physical Health Issues
		Addiction and Dependence

### Sensory and experiential appeal

Within this theme, participants described both internal bodily sensations and external sensory engagement. Pleasant physical sensations refer to internal experiences such as cooling, lightheadedness, and relaxation, whereas playing with vapor/smoke reflects the visual, tactile, and playful interaction with vapor production. This distinction helps clarify the different dimensions of sensory appeal reported by users. This theme emerged from the following sub-themes:

#### Curiosity and novelty

This dimension refers to the individual’s desire to experience new things, including e-cigarettes. In many cases, the initial use was driven by pure curiosity to “see what it’s like” or “understand why others use it.”

*I tried vaping out of curiosity. I wanted to know what feeling it gives.”* (Participant, 25 years old).

#### Pleasant physical sensations

Descriptions such as “a cooling sensation” in the brain and throat or “it nicely makes your head spin” indicate a distinct physical sensation that users find enjoyable.

*Your brain and throat feel cool*,* and your mind feels cleared.” (Participant*,* 24 years old)*.

#### Pleasant flavors and flavor variety

The presence of a “good taste,” “the taste of its essence,” and the desire to “try all the flavors” indicate that flavor variety and palatability are significant attractions. Users described them as “tasty and enjoyable,” noting that they do not leave a bitter taste in the mouth.

*I want to try all its flavors; each one appeals to you differently.” (Participant*,* 24 years old)*.

#### Playing with vapor/smoke

The act of exhaling vapor was described as “enjoyable and ephemeral,” pointing to visual and sensory satisfaction.

*“You can play with its vapor; it’s not like cigarette smoke that irritates your eyes.” (Participant*,* 25 years old)*.

### Emotional regulation and stress relief

Many participants used e-cigarettes as a mechanism to manage negative emotional states, indicating a perceived calming or distracting effect. This theme consists of the following sub-categories:

#### Coping with negative emotions

Users often turned to e-cigarettes when feeling “angry,” “upset,” “stressed,” or “anxious,” finding that they “provide calmness” or help them relax.

*When I’m stressed*,* it calms me down and helps me relax.” (Participant*,* 21 years old)*.

#### Accompanying intense emotions

Another concept brought up by users was consuming e-cigarettes during intense emotional situations, including positive ones. Use was reported during exciting events like watching a thrilling football match, group gatherings with friends, or watching an impactful movie, indicating that consumption occurs alongside both negative and highly positive emotions.

*I use it when I feel very good or very sad.” (Participant*,* 28 years old)*.

### Social contexts and normalization

The social environment plays a crucial role in both the initiation and continuation of e-cigarette use, with peers, family, and public perception strongly influencing this behavior. This theme consists of the following sub-categories:

#### Peer pressure and modeling

Many users started consuming e-cigarettes after seeing their friends use them or being encouraged by them, particularly in social gatherings. Presence in these groups creates a form of social pressure, where adopting group behavioral norms, including e-cigarette use, is perceived as necessary for social acceptance.

*I saw it in my friends’ hands*,* and when they offered*,* I tried it too.” (Participant*,* 29 years old)*.

#### Social acceptance and normalization

Vaping is increasingly perceived as “normal and natural,” with users believing that no one objects to it. This could be due to a lack of public awareness about its harms or its distinct appearance, which differs from traditional cigarettes and lacks their negative imagery.

*Vaping has become so normal that no one really objects to it.” (Participant*,* 25 years old)*.

#### Vaping as a status symbol

Use of e-cigarettes by influencers contributes to a perception of them being “classy” or prestigious.

*“When influencers use it*,* it looks classy and modern.” (Participant*,* 22 years old)*.

#### Family acceptance and modeling

Interestingly, some users noted that their families, who might oppose traditional cigarettes, were more accepting of e-cigarettes. This acceptance could be influenced by existing family traditions, such as hookah smoking, or even family members trying e-cigarettes themselves. Given that hookah use is part of the social and cultural tradition in Bandar Abbas, especially among older generations, acceptance of e-cigarettes is easier within families already familiar with hookah consumption.

*“My family doesn’t mind vaping because they think it’s like fruit-flavored hookah.” (Participant*,* 21 years old)*.

#### Lack of recreational spaces

This sub-category indicates that some consumers were inclined towards e-cigarette use due to the lack of suitable and attractive recreational facilities for factors influencing e-cigarette use among adults aged 19–52 years people.

*“We vape because there’s no other entertainment when we go out.” (Participant*,* 22 years old)*.

### Perceived practical benefits and misconceptions

Users often justified e-cigarette use based on perceived advantages over traditional cigarettes. A common belief among consumers is that this type of cigarette is less harmful than conventional ones. They perceive the produced vapor as less heavy and, because it is generated in a moist chamber, believe it has fewer side effects.

#### Less nicotine

A common belief is that e-cigarettes are “much less harmful than cigarettes,” have “very little nicotine,” and can even be useful for “quitting smoking.”

*“People say it has very little nicotine and is better than cigarettes.” (Participant*,* 24 years old)*.

#### Vapor instead of smoke

Some consumers believed that vapes do not produce smoke but rather a very light vapor [aerosol] that quickly dissipates in the air, making it less harmful. Their point was that, because this type of cigarette contains more moisture than conventional dry cigarettes, it causes less damage to the throat, teeth, and lungs. This perception is similar to the image some hold of hookah being less harmful.

*“It feels like steam and doesn’t bother my throat.” (Participant*,* 21 years old)*.

#### Tool for smoking cessation

This concept reveals a strange belief among some consumers that by using e-cigarettes, they can easily quit conventional cigarettes and avoid their harms. The repetition of this concept among participants indicates that this claim has been seriously propagated within the consumer community and has become a firm belief.

*• “I thought vaping would help me quit*,* but now I use both.” (Participant*,* 25 years old)*.

### The role of the tobacco industry

In this study, the term “tobacco industry” is used broadly to describe the network of actors involved in the promotion, distribution, and commercialization of e-cigarettes. This includes not only formal tobacco-related companies but also informal vendors, online sellers, social media influencers, advertising platforms, and other stakeholders who financially benefit from vaping-related products. These actors collectively contribute to increasing the accessibility, attractiveness, and normalization of e-cigarette use.

#### Portability

E-cigarettes are easily portable and can be carried anywhere. Vapes are designed in a way that some ordinary people don’t recognize them, avoiding the creation of a negative or misleading appearance.

*“I can take it anywhere without anyone noticing.” (Participant*,* 27 years old)*.

#### Affordability

Some users considered vaping to be much more cost-effective than traditional cigarettes.

*“Vaping costs less than regular cigarettes.” (Participant)*.

#### Aesthetic appeal

The physical appearance of the device itself can be attractive, with users finding it “very cool” or “very pleasing.”

*“I really liked its design; it looked very cool.” (Participant*,* 23 years old)*.

### Physical and psychological complications

Although many believe in the benefits, there are also negative stories. Most of these involved physical and psychological issues. Some consumers also reported psychological difficulties such as severe dependence and regret about use.

#### Physical health issues

The most significant challenges reported by participants included: stomach pain, shortness of breath, tooth decay, and lung problems.

*“At first I thought it was better*,* but it damaged my teeth and throat.” (Participant*,* 24 years old)*.

#### Addiction and dependence

- Participants demonstrated a clear recognition of the highly addictive nature of both cigarettes and vapes, describing an overwhelming and persistent craving that dominated their daily lives. They reported experiencing a constant desire to use these products, accompanied by feelings of loss, emptiness, and anxiety when unable to consume them, illustrating the powerful psychological grip of nicotine dependence.

*“When you don’t use it*,* you feel like you’ve lost something.” (Participant*,* 27 years old)*.

## Discussion

This qualitative study is among the first to examine factors influencing e-cigarette use among adults aged 19–52 years in Bandar Abbas, a context characterized by cultural normalization of hookah use and formal restrictions on e-cigarettes. Interpreted through a social-ecological and cultural lens, the findings demonstrate that vaping behavior is not driven by a single factor but emerges from the interaction of sensory, emotional, social, economic, and regulatory influences operating across sensory, emotional, social, economic, and regulatory influences.

Sensory and experiential appeal emerged as the dominant driver of both initiation and continuation of vaping. While prior studies have consistently documented the role of flavors, curiosity, and vapor production in motivating use [2, 26], the present study extends this literature by showing that these sensory drivers remain powerful even in a regulatory environment where e-cigarettes are officially banned. This suggests that sensory gratification functions as a regulatory-resistant mechanism of use, reinforcing vaping behavior independently of legal constraints. From a public health perspective, this finding highlights a critical limitation of policy approaches that focus primarily on supply restriction without addressing the experiential appeal of products.

A novel contribution of this study is the explicit identification of “vapor play” as a recreational practice embedded in local cultural norms. Unlike Chen et al. (2019), who emphasized flavor appeal with limited attention to recreational vapor production [27], participants in Bandar Abbas described vapor production as a form of visual and social entertainment. This pattern appears closely linked to the cultural legacy of hookah use, where smoke visibility and performance are socially valued [22]. Situated within a cultural-behavioral framework, this finding suggests that vaping in this context is not merely a nicotine delivery behavior but a culturally adapted leisure activity, aligning with but also extending international qualitative findings [28, 29].

Emotional regulation was another central theme, with participants describing vaping as a means of coping with stress and negative affect, as well as enhancing positive emotional and social experiences. Consistent with prior qualitative research [30–33], vaping functioned as an affect-regulation strategy. However, unlike studies that emphasize vaping primarily as a response to distress [34], participants in this study frequently reported use in positive, collective settings such as social gatherings and leisure activities. This distinction underscores the importance of cultural context, where shared emotional experiences and social rituals may normalize and reinforce vaping behavior beyond individual stress management [22]. From a social-ecological perspective, emotional regulation through vaping appears to be shaped not only by individual psychological needs but also by socially patterned leisure practices.

Social normalization processes were particularly salient in this setting. While peer influence, social media exposure, and influencer marketing are well-documented globally [35–38], this study highlights the distinctive role of family tolerance rooted in the cultural acceptability of hookah smoking. In contrast to studies that marginalize familial influence [39], family acceptance in Bandar Abbas appears to lower perceived risk and social barriers to vaping. Additionally, participants linked vaping uptake to a lack of alternative recreational spaces, a structural factor less frequently emphasized in Western contexts [40, 41]. These findings illustrate how environmental and infrastructural constraints interact with social norms to shape health-related behaviors in mid-sized cities.

Misconceptions regarding the perceived benefits of e-cigarettes—such as reduced harm and smoking cessation efficacy—were widespread and strongly reinforced by industry messaging. Although these beliefs align with earlier studies [26, 36], the particularly strong endorsement of vaping as a cessation tool contrasts with more skeptical accounts in recent literature [42]. This divergence likely reflects weaker public health communication and regulatory oversight in Iran compared to settings with sustained anti-vaping campaigns [43]. Within a public health framework, this finding emphasizes how regulatory gaps and marketing practices can amplify misinformation and shape risk perceptions in vulnerable populations.

Economic and product-related factors further reinforced vaping behavior. While device design and portability mirror global industry strategies [44, 45], the emphasis on affordability represents a context-specific driver shaped by Iran’s economic conditions. Unlike Rutherford et al. (2023), who downplayed cost considerations [46], participants in this study explicitly framed vaping as a financially accessible alternative to cigarettes. This highlights how macroeconomic factors intersect with individual decision-making in low- and middle-income settings [38].

Finally, the progression from experimentation to dependence and regret illustrates the long-term consequences of vaping behavior. Although addiction and regret have been documented previously [41, 42], participants in this study reported a broader range of physical symptoms, including respiratory and gastrointestinal problems. This contrasts with studies emphasizing primarily emotional regret [27] and may reflect differences in product quality or regulatory oversight, as suggested by Heckman et al. (2019) [47]. These findings underscore the need for localized surveillance of health effects and stronger regulatory enforcement.

Taken together, this study advances existing literature by demonstrating how globally recognized drivers of vaping are reconfigured through local cultural norms, economic conditions, and regulatory environments. By situating findings within a social-ecological and cultural framework, the study moves beyond replication to illustrate how vaping behaviors are shaped by context-specific interactions across multiple levels. These insights have important implications for public health interventions, suggesting that effective prevention strategies must address not only individual behavior but also cultural practices, social norms, and structural constraints.

### Strengths and limitations

This study has several limitations that should be considered when interpreting the results. First, data collection was confined to the city of Bandar Abbas; therefore, generalizing the findings to other regions should be done cautiously. Second, the data were obtained through participant self-report, which introduces the potential for recall bias or a tendency to provide socially desirable responses. Third, given the qualitative nature of the study, the aim was to identify patterns and meanings rather than to estimate frequencies or test hypotheses. Lastly, we did not collect data on participants’ opinions regarding e-cigarette restrictions. Given Iran’s complete ban on e-cigarettes, we were concerned that discussing regulatory perspectives might inhibit candid responses about personal use. However, future studies should explore users’ perceptions of tobacco control policies from broader perspectives, particularly given the tension between harm reduction approaches for adult smokers versus harm prevention strategies for youth.

## Conclusion

Our findings reveals that e-cigarette use in Bandar Abbas is a multidimensional phenomenon driven by sensory appeal, psychological functions, social normalization, and widespread misconceptions about safety. The interplay of individual factors, such as diverse flavors and stress relief, with social influences including peer pressure and influencer impact, has rendered vaping commonplace and even prestigious in some circles. Critically, industry marketing has successfully promoted misconceptions about reduced harm and smoking cessation efficacy, masking the reality of respiratory problems, severe dependency, and user regret reported by participants. These findings underscore the urgent need for multilevel, culturally tailored interventions in Iran. Beyond public awareness campaigns to correct misconceptions, efforts must include stricter regulation of the tobacco industry advertising, development of healthy recreational alternatives for youth, and strengthening of stress-coping skills. The observed patterns, shaped by local hookah traditions, economic accessibility, and limited leisure infrastructure (especially for youngsters), highlight the importance of context-specific approaches and cross-cultural research to inform effective policy responses in low and middle-income countries like Iran.

### Practical Implications

The findings of this research can guide the design of preventive interventions and policies for controlling electronic cigarette use in similar cultural contexts. Educational programs should focus on correcting misconceptions, particularly regarding “reduced harm” and “aid in smoking cessation.” Social interventions can be more effective by mitigating peer influence, restricting advertising and online sales, and creating healthy alternatives for youth leisure activities. It is also recommended that health messages be tailored to the language and lived experiences of the target groups to maximize their impact.

## Supplementary Information

Supplementary Material 1.

## Data Availability

All extracted data used in this review has been reported in the text, figures and tables (including Appendices).
