# Supplementary material for: E-cigarette use among adults in southern Iran: a qualitative study
Source: BMC Public Health. 2026 Jun 9;26:2346. doi: 10.1186/s12889-026-27917-w (PMC13471470; doi:10.1186/s12889-026-27917-w)
Supplement: Supplementary file 1 [file 12889_2026_27917_MOESM1_ESM.pdf]

Title of Study

E-cigarette use among adults in southern Iran: A qualitative study

(Experiences, Attitudes, and Perspectives of Adults Regarding the Use of Electronic Cigarettes (E-Cigarettes) in Southern Iran)

Purpose of the Interview

The purpose of this interview is to explore and understand adults' experiences, attitudes, and perceptions regarding the use of electronic cigarettes. The interview aims to identify the reasons for initiating and continuing use, perceived physical and psychological effects, and personal views on the advantages and disadvantages of e-cigarette use within the social and cultural context of southern Iran.

Demographic Information of the Participant

(To be completed by the interviewer)

Interview Code: ....

Gender: ☐ Male ☐ Female

Age: ....

Marital Status: ....

Education Level: ....

Occupation: ....

History of Conventional Cigarette Use: ☐ Yes ☐ No

Duration of E-Cigarette Use: ....

Interview Procedure

This is a semi-structured, in-depth interview conducted face-to-face. Participants are encouraged to express their experiences and opinions freely. The interviewer may ask probing or follow-up questions based on participants' responses to gain deeper insight into the topic. Each interview is expected to last approximately 30 to 70 minutes.

Main Interview Questions

1) Could you please describe the first time you used an e-cigarette?
2) What factors or circumstances led you to start using it?
3) How would you describe your current pattern of e-cigarette use?
4) How often and under what conditions do you usually use it?
5) From your perspective, what are the advantages and disadvantages of using e-cigarettes?
6) What physical or psychological effects have you experienced since you started using e-cigarettes?
7) Have you ever compared e-cigarettes with traditional cigarettes?
8) In your opinion, what are the similarities and differences?
9) How do your family members, friends, or society perceive your use of e-cigarettes?
10) Are you using e-cigarettes as a way to quit smoking traditional cigarettes, or do you have other reasons for continuing to use them?
11) What barriers or challenges have you encountered in using e-cigarettes?
12) What advice or recommendations would you give to individuals who are considering starting or quitting e-cigarette use?

Interviewer's Notes

(Non-verbal observations, contextual notes, and initial reflections)

....

....

....

Ethical Considerations

Prior to the interview, informed consent will be obtained from each participant. Participants will be assured that all information shared will remain confidential and will be used solely for research purposes. Participation is voluntary, and individuals may withdraw from the interview at any time without any consequences.
